# Pir-B inhibits the DC function and disturbs the Th17/Treg balance in lung cancer murine model

**DOI:** 10.18632/oncotarget.21763

**Published:** 2017-10-10

**Authors:** Huaxing Wu, Xiaoyu Zheng, Linlin Dong, Chunfeng Li, Mingyue Zhang, Guonian Wang, Kun Wang

**Affiliations:** ^1^ Department of Anesthesiology, Harbin Medical University Cancer Hospital, Harbin 150081, China

**Keywords:** Pir-B, Th17, Treg, immune, lung cancer

## Abstract

Paired immunoglobulin-like receptor B (Pir-B) was an inhibitory receptor expressed on the surfaces of dendritic cells (DCs). Pir-B inhibit T helper (Th) 1 response and induce Th2 cell differentiation, leading to the imbalance of Th1/Th2 cells. However, the role and potential mechanism of Pir-B on the balance of Th17/regulatory T cells (Tregs) is still largely unknown in lung cancer murine model. In the present study, the DC function and Th17/Treg balance were destroyed during the progression of lung cancer and this was accompanied by an increased expression of Pir-B. After transfection with Pir-B siRNA or administration of IL-6 *in vitro,* the decreased response of Th17 cells were restored, whereas the augmented differentiation of Tregs was diminished. Further, the transfer of Pir-B silenced DCs or the injection of IL-6 *in vivo* increased Th17 response and decreased Treg differentiation. Our study has demonstrated that Pir-B inhibits the DC function and disturbs the Th17/Treg balance via IL-6 pathway during the progression of lung cancer, contributing to inhibited antitumor immunity.

## INTRODUCTION

Lung cancer is the leading cause of cancer-related death in China with a tendency to metastasize widely during the course of the disease [1]. Lung tumors escape because the anti-tumor immunity itself was suppressed, and the impaired immune surveillance is inclined to tumor cells proliferation and metastasis [2]. Studies suggest that the adaptive immune system is identified as an indispensable participant of tumor immune pathogenesis [3, 4]. There is a need, therefore, to elucidate the immunosuppressive mechanism underlying in the progression of lung cancer to develop new immunotherapy strategies.

As the important components of adaptive immune system, CD4^+^ T cell subsets initiate different immune responses, and the balance between antitumor immunity and tumor immune evasion determines the direction of the malignant process [5–7]. Activated CD4^+^ T cells differentiate into Th1, Th2, Th17, and regulatory T cells (Tregs) based on the secretion of cytokines. Previous studies indicated that Th1 cells augmented antitumor responses, whereas Th2 cells downregulated antitumor immunity, and the balance of Th1/Th2 was disturbed during the progression of lung cancer, contributing to tumor related immunosuppression [8]. Recently, the Th17/Treg balance has been considered to be essential for maintaining the immune steady state [9, 10]. Tregs are potent suppressors of antitumor immunity [11]. However, the role of IL-17 in tumor immunity remains undefined. IL-17 deficiency or IL-17 blockade led to suppression of lung metastasis in tumor model, indicating that IL-17-mediated responses promotes tumor development [12]. In contrast, tumor growth in subcutaneous tissue and lung tumor metastasis are enhanced in IL-17^−/−^ mice [13, 14]. It implicates that IL-17-mediated response is protective against tumor development [2]. However, the roles of Th17 cells and Tregs in lung cancer immunopathology remain largely unknown, and targeting the Th17/Treg balance may be useful for lung cancer treatment.

DCs are key antigen presenting cells (APCs) controlling the initiation and differentiation of CD4^+^ T cells [15]. In mediating these roles, DCs pass through different functional states [16]. Immature DCs (imDCs) in the resting state are highly efficient in the capture and uptake of antigens, and mature DCs (mDCs) in the activated state possess the strongest antigen processing and presenting ability and have high expression levels of co-stimulatory molecules on their surfaces [15]. mDCs can activate the responses of Th1 and Th17 cells, and imDCs promote the differentiation of Th2 and Treg cells [17]. Recently, paired immunoglobulin like receptor B (Pir-B), an inhibitory receptor expressed on the surface of DCs, inhibit the DC maturation and Th1 response and induce Th2 cell differentiation [18–20]. Above studies provide a clue that Pir-B down-regulated the DC maturation and indirectly regulate the differentiation of CD4^+^ T cell subsets. Therefore, in the present study, the role and potential mechanism of Pir-B on the DC function and Th17/Treg balance were investigated in a Lewis lung cancer (LLC) murine model.

## RESULTS

### *In vitro* cytotoxicity assessment

**Figure 1:**
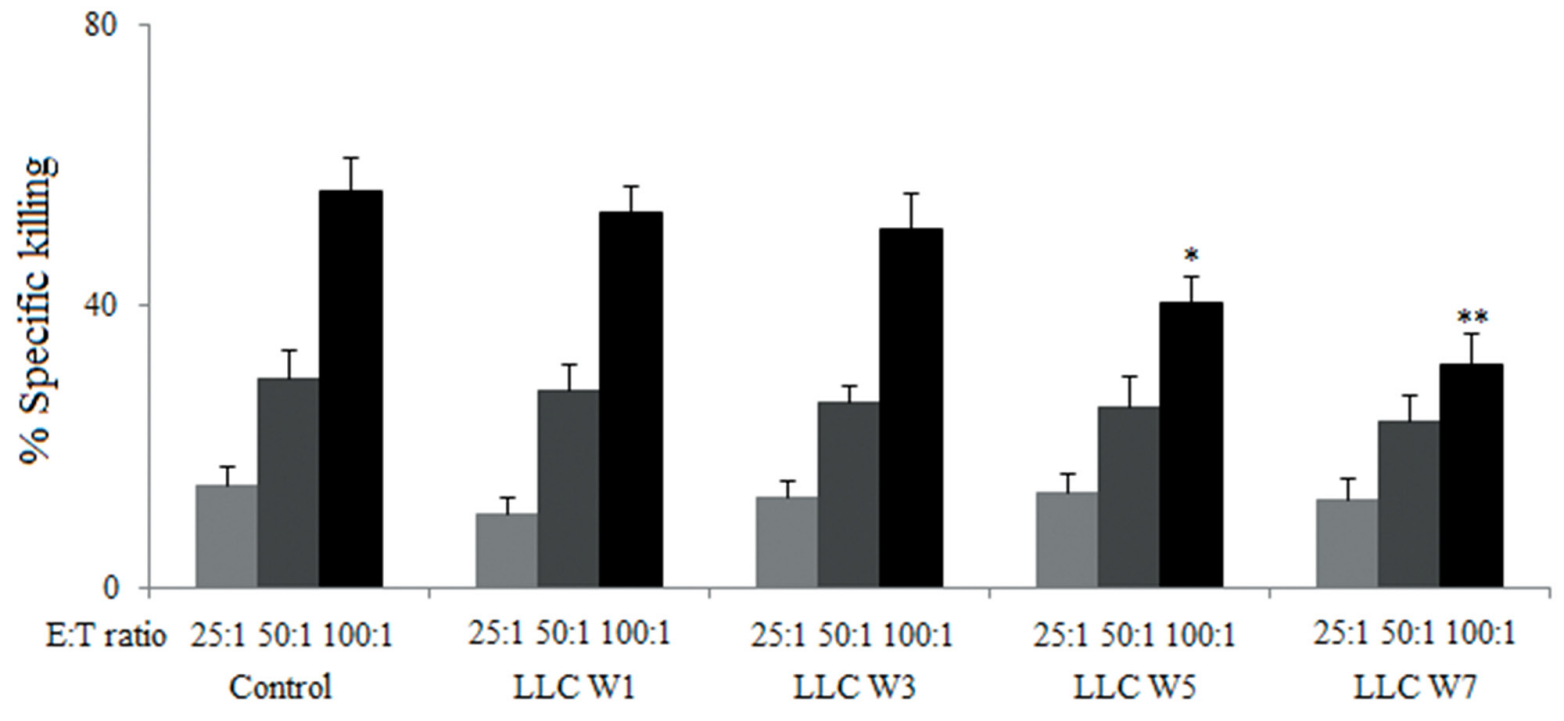
The cytotoxicity of splenic lymphocytes were inhibited by tumor cells Splenic lymphocytes were incubated with LLC cells (2×10^5^/well) at different effector/target ratios (25:1, 50:1 and 100:1) in the 96-well culture plates for 4 h. The supernatants were collected and the absorbance at 490 nm was tested to evaluate the lactate dehydrogenase (LDH). Control (Tumor-free mice, n=10), LLC W1, 3, 5 and 7 (1, 3, 5 and 7 weeks after LLC inoculation, n=10/group). The results are shown as the means ± SD of three different experiments. ^*^P<0.05, ^**^P<0.01 versus Control.

The cytotoxicity of splenic lymphocytes was significantly inhibited at 5 and 7 weeks after LLC inoculation in tumor-bearing mice compared with Control at the effector/target ratio of 100:1 (Figure 1). Meanwhile, no statistical significance was found among groups at the effector/target ratios of 25:1 and 50:1. These results suggested that tumor cells inhibited the cytotoxicity of splenic lymphocytes.

### The Th17/Treg balance was disturbed during the progression of lung cancer

**Figure 2:**
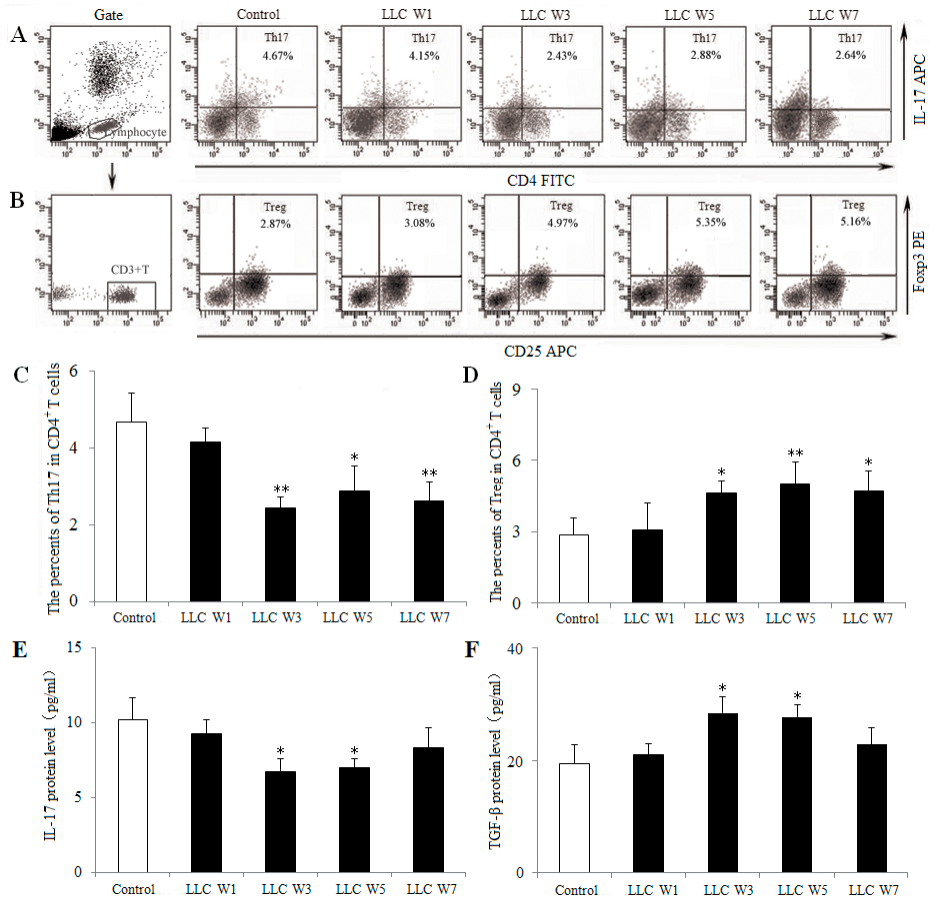
The Th17/Treg balance was disturbed during the progression of lung cancer Single cell suspensions from tumor tissues were stained with labeled antibodies as described in Materials and Methods. The percentages of Th17 **(A)** and Treg **(B)** cells in CD4^+^ T cells were measured by flow cytometry. The percentages of Th17 **(C)** and Treg **(D)** cells were shown in the histogram. The protein levels of IL-17 **(E)** and TGF-β **(F)** were assayed by ELISA using the homogenate of tumor tissues. Control (Tumor-free mice, n=10), LLC W1, 3, 5 and 7 (1, 3, 5 and 7 weeks after LLC inoculation, n=10/group). The results are shown as the means ± SD of three different experiments. ^*^P<0.05, ^**^P<0.01 versus Control.

To characterize the Th17/Treg balance, the percentages of Th17 cells and Tregs infiltrating in tumor tissue were examined at 1, 3, 5 and 7 weeks after LLC inoculation. The percentage of Th17 cells (CD4^+^ IL-17^+^) in tumor-bearing mice was significantly lower, whereas the percentage of Tregs (CD25^+^ Foxp3^+^) in tumor-bearing mice was higher at 3, 5 and 7 weeks after LLC inoculation than that in Control (Figure 2A–2D). These data confirmed that the Th17/Treg balance was disturbed during the progression of lung cancer, and this imbalance lead to tumor related immunosupression.

To further elucidate the Th17/Treg balance, related cytokines were detected by ELISA using the homogenate of tumor tissues. IL-17 protein level was down-regulated, whereas TGF-β protein level was up-regulated at 3 and 5 weeks after LLC inoculation compared with Control (Figure 2E and 2F). These findings provided further evidence a shift in the Th17/Treg balance toward Treg differentiation and Th17 response was inhibited during the progression of lung cancer.

### The function and maturation of DCs were inhibited during the progression of lung cancer

**Figure 3:**
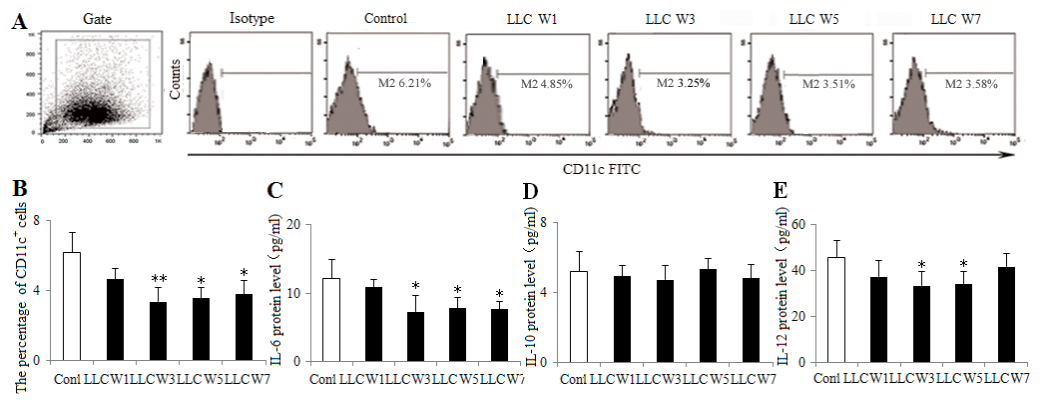
Changes in the DC function during the progression of lung cancer Single cell suspensions from tumor tissues were stained with CD11c^+^ antibody. The percentage of DCs **(A)** was analyzed by flow cytometry. The percentage of DCs **(B)** was shown in the histogram. The protein levels of IL-6 **(C)**, IL-10 **(D)** and IL-12 **(E)** were measured by ELISA using the homogenate of tumor tissues. Control (Tumor-free mice, n=10), LLC W1, 3, 5 and 7 (1, 3, 5 and 7 weeks after LLC inoculation, n=10/group). The results are shown as the means ± SD of three different experiments. ^*^P<0.05, ^**^P<0.01 versus Control.

To understand the DC function, the percentage of DCs infiltrating in tumor tissues was measured at 1, 3, 5 and 7 weeks after LLC inoculation. DCs were defined by the expression of CD11c, an established marker. The percentage of DCs was lower at 3, 5 and 7 weeks after LLC inoculation in tumor-bearing mice than that in Control (Figure 3A and 3B). The levels of related cytokines (IL-6, IL-10 and IL-12) were also detected by ELISA using the homogenate of tumor tissues. The protein levels of IL-6 at 3, 5 and 7 weeks after LLC inoculation and IL-12 at 3 and 5 weeks after LLC inoculation were decreased in tumor-bearing mice compared with Control (Figure 3C and 3E), whereas IL-10 protein level did not differ among groups. These results supported the notion that the DC function was inhibited during the progression of lung cancer.

**Figure 4:**
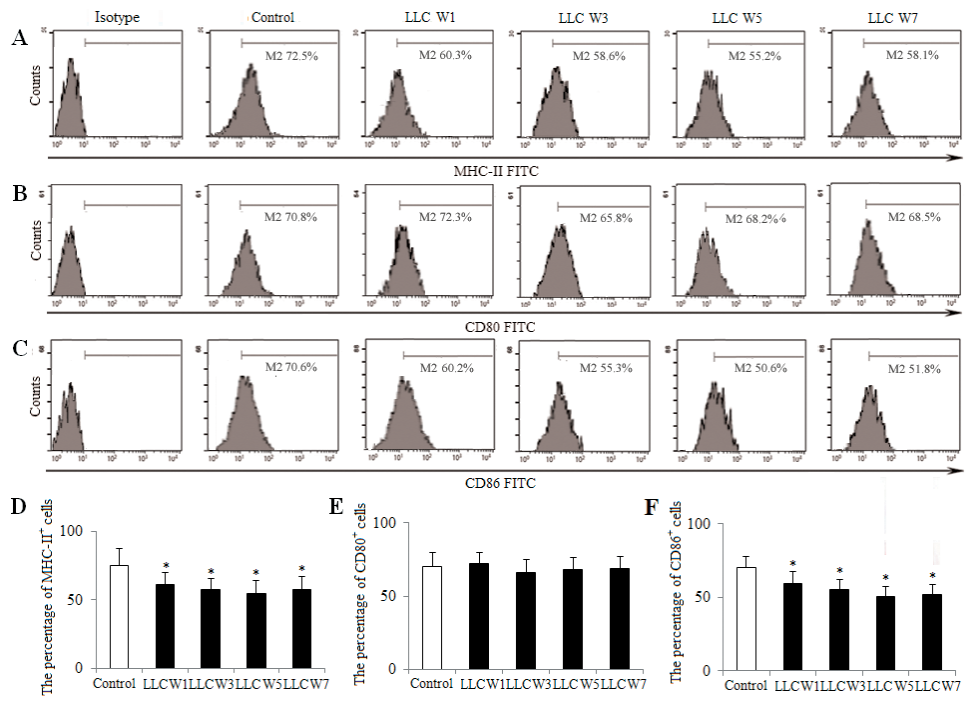
Changes in the DC maturation during the progression of lung cancer Single cell suspensions from tumor tissues were stained as described in Materials and Methods. The percentages of MHC-II **(A)**, CD80 **(B)** and CD86 **(C)** were analyzed by flow cytometry. The percentages of MHC-II **(D)**, CD80 **(E)** and CD86 **(F)** were shown in the histogram. Control (Tumor-free mice, n=10), LLC W1, 3, 5 and 7 (1, 3, 5 and 7 weeks after LLC inoculation, n=10/group). The results are shown as the means ± SD of three different experiments. ^*^P<0.05, ^**^P<0.01 versus Control.

Next, to further investigate the DC maturation, the expression of MHC-II, CD80 and CD86 on the surfaces of DCs was assayed. The percentages of MHC-II and CD86 in tumor-bearing mice were significantly decreased at 1, 3, 5 and 7 weeks after LLC inoculation compared with Control (Figure 4), whereas the percentage of CD80 was not statistically different. These observations indicated that the DC maturation was inhibited, as indicated by attenuating the expression of MHC-II and CD86 during the progression of lung cancer.

### The increased expression of Pir-B inhibited the DC function and disturbed the Th17/Treg balance

**Figure 5:**
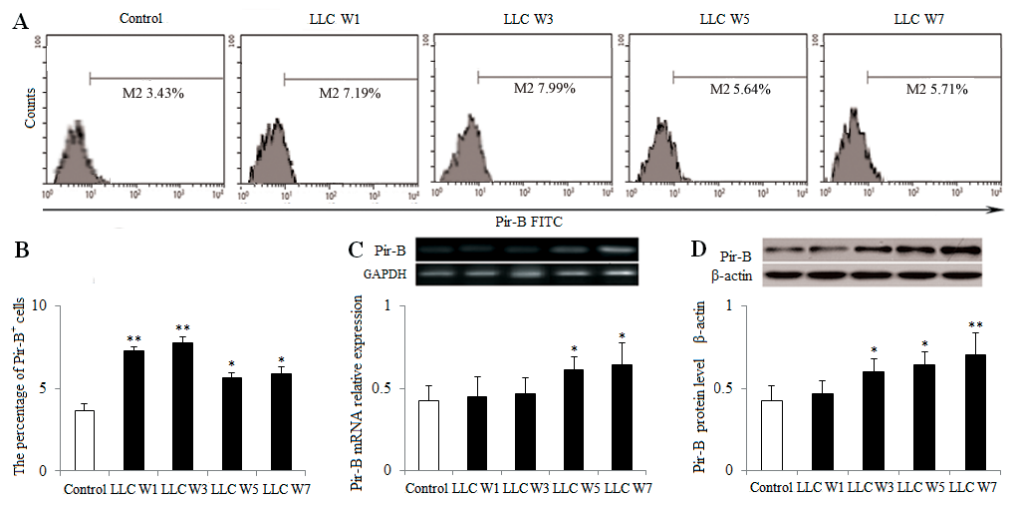
Changes in the expression of Pir-B during the progression of lung cancer Single cell suspensions from tumor tissues were stained. **(A)** The expression of Pir-B was analyzed by flow cytometry. **(B)** The percentage of Pir-B was shown in the histogram. The levels of Pir-B mRNA **(C)** and protein **(D)** were detected by PCR and Western blotting, respectively. The data are representative of three independent experiments. Control (Tumor-free mice, n=10), LLC W1, 3, 5 and 7 (1, 3, 5 and 7 weeks after LLC inoculation, n=10/group). The results are shown as the means ± SD of three different experiments. ^*^P<0.05, ^**^P<0.01 versus Control.

To definite the role of Pir-B, the expression of Pir-B was estimated at 1, 3, 5 and 7 weeks after LLC inoculation. The percentage of Pir-B in tumor-bearing mice was increased at 1, 3, 5 and 7 weeks after LLC inoculation compared with Control (Figure 5A and 5B). The levels of Pir-B mRNA at 5 and 7 weeks after LLC inoculation and protein at 3, 5 and 7 weeks after LLC inoculation were significantly increased in tumor-bearing mice compared with Control (Figure 5C and 5D). These results suggested that the expression of Pir-B was increased during the progression of lung cancer.

**Figure 6:**
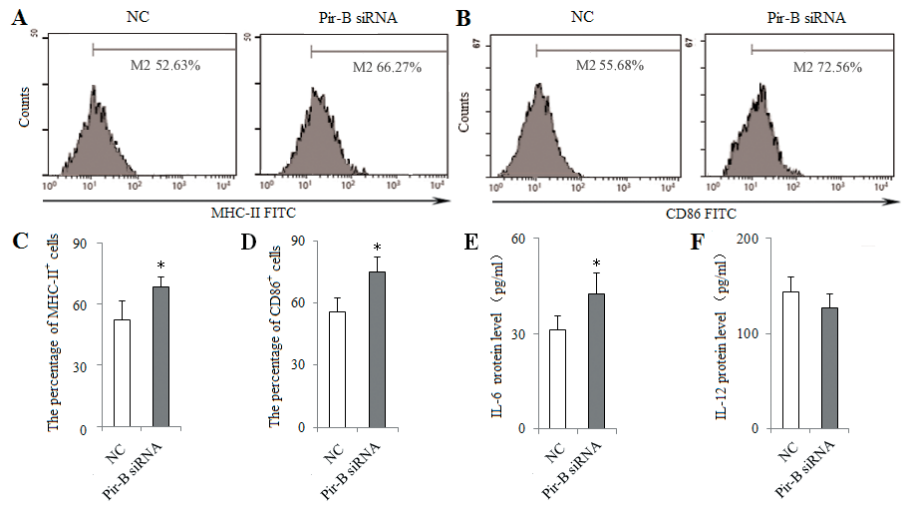
Knockdown of Pir-B by specific siRNA increased the DC function DCs isolated from spleen of 5-week tumor-bearing mice were transfected with either negative control (NC) siRNA or Pir-B siRNA and co-cultured with CD4^+^ T cells. After 3 days of transfection, the percentages of MHC-II **(A)** and CD86 **(B)** were determined by flow cytometry. The percentages of MHC-II **(C)** and CD86 **(D)** were shown in the histogram. The protein levels of IL-6 **(E)** and IL-12 **(F)** in the supernatant of co-cultures were assessed using ELISA. The data are representative of three independent experiments. ^*^P<0.05, ^**^P<0.01 versus NC.

**Figure 7:**
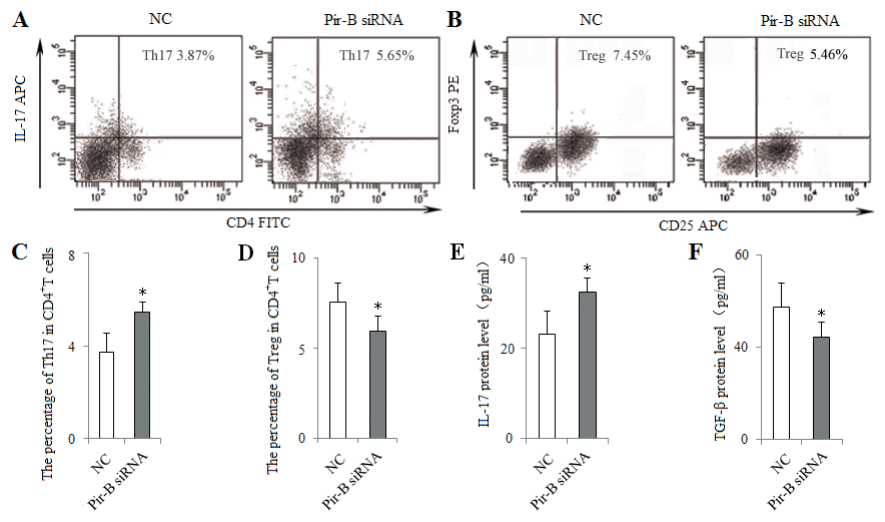
*In vitro* knockdown of Pir-B increased Th17 response and decreased Treg differentiation DCs isolated from spleen of 5-week tumor-bearing mice were transfected with either negative control (NC) siRNA or Pir-B siRNA and co-cultured with CD4^+^ T cells. After 3 days of transfection, the percentages of Th17 **(A)** and Treg **(B)** cells were analyzed by flow cytometry. The percentages of Th17 **(C)** and Treg **(D)** cells were shown in the histogram. The protein levels of IL-17 **(E)** and TGF-β **(F)** in the supernatant of co-cultures were assessed using ELISA. ^*^P<0.05, ^**^P<0.01 versus NC.

To block the role of Pir-B *in vitro*, DCs isolated from 5-week tumor-bearing mice were transfected with siRNA Pir-B, and co-cultured with CD4^+^ T cells. First, the inhibitory efficiency of Pir-B *in vitro* was estimated after transfection, Pir-B siRNA significantly reduced the mRNA and protein levels of Pir-B. The apparent decrease (69.6%) was observed at 3 days after transfection. Next, the higher levels of MHC-II, CD86 and IL-6 were observed in Pir-B siRNA group with negative control (NC) group (Figure 6), whereas the difference in the level of IL-12 was not significant. The inhibited expression of Th17 cells and IL-17 was restored, whereas the augmented expression of Tregs and TGF-β was diminished in Pir-B siRNA group compared with NC group (Figure 7). These findings suggest that Pir-B inhibited the DC function and disturbed the Th17/Treg balance.

**Figure 8:**
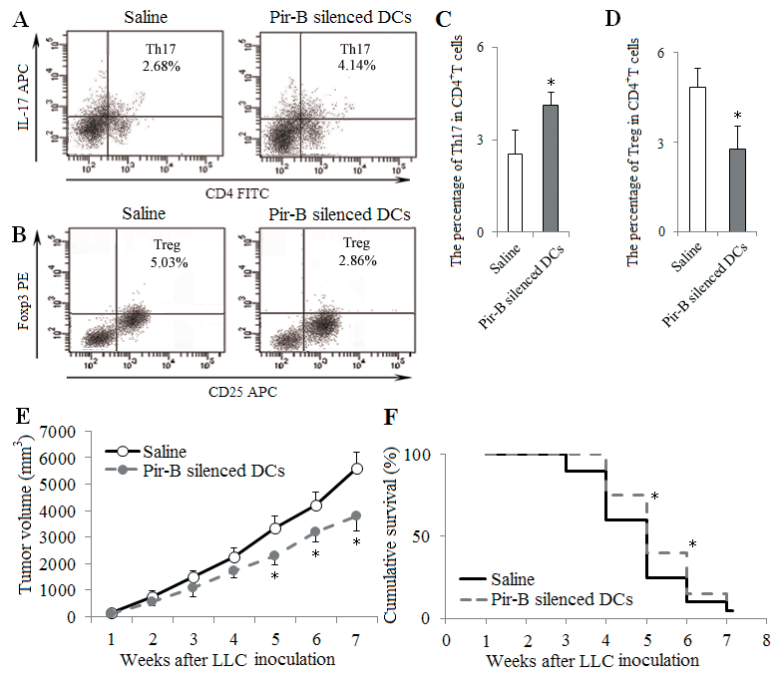
*In vivo* transfer of Pir-B silenced DCs increased Th17 response and decreased Treg differentiation DCs transfected with Pir-B siRNA (1 × 10^6^ cells per mice) were transferred into recipient animals by tail vein injection at 1, 2 and 3 weeks after LLC inoculation. Saline (0.1ml) was considered as control. The percentages of tumor infiltrating Th17 **(A)** and Tregs **(B)** cells were measured. The percentages of Th17 **(C)** and Treg **(D)** cells were shown in the histogram. Tumor growth **(E)** was monitored once weekly, and tumor volume was assessed by measuring tumor size with digital calipers. Ten mice from each group were randomly selected for the survival rate analysis **(F)**. ^*^P<0.05, ^**^P<0.01 versus Saline.

To block the role of Pir-B *in vivo*, Pir-B silenced DCs (1 × 10^6^ cells per mice) were transferred into tumor-bearing mice by tail vein injection at 1, 2 and 3 weeks after LLC inoculation. The percentage of tumor infiltrating Th17 cells was increased, whereas the percentage of tumor infiltrating Tregs was decreased in mice treated with Pir-B silenced DCs compared with Saline group (Figure 8A–8D), As shown in Figure 8E, tumor cells showed progressive growth, and tumors were palpable at 1 week after LLC inoculation. Average tumor volumes in tumor-bearing mice were 3350±450 mm^3^ at 5 weeks after LLC inoculation in Saline group, and a significant reduction in tumor volume was observed at 5, 6 and 7 weeks after LLC inoculation in mice treated with Pir-B silenced DCs compared with Saline group. As shown in Figure 8F, there was a significant improvement in the survival rate of mice treated with Pir-B silenced DCs at 5 and 6 weeks after LLC inoculation than that in Saline group. These *in vivo* data suggest that Pir-B was involved in the imbalance of Th17/Tregs, and tumor growth, and the survival rate of tumor-bearing mice.

### Pir-B disturbed the Th17/Treg balance via IL-6 pathway

**Figure 9:**
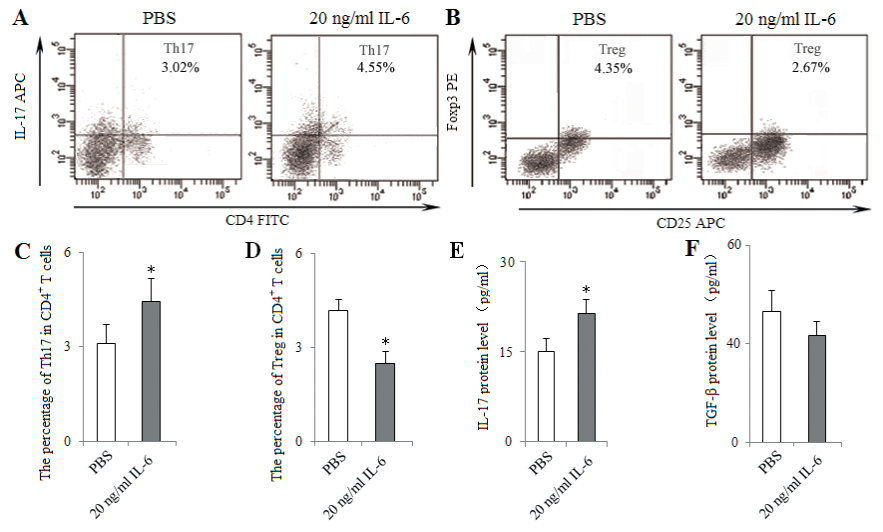
*In vitro* administration of IL-6 induced Th17 response and inhibited Treg differentiation IL-6 was administered at the optimal dose (20 ng/ml) in a co-culture of CD4^+^ T cells and DCs isolated from spleen of 5-weeks tumor-bearing mice at the ratio of 10:1, 2.5 μg/ml anti-CD28 mAb in anti-CD3 mAb-coated (5 μg/ml) 96-well plates. PBS (0.1ml) was considered as control. After 48 h of co-culture, the percentages of Th17 **(A)** and Treg **(B)** cells were analyzed with flow cytometry. The percentages of Th17 **(C)** and Treg **(D)** cells were shown in the histogram. The protein levels of IL-17 **(E)** and TGF-β **(F)** in the supernatant of co-cultures were assessed using ELISA. ^*^P<0.05, ^**^P<0.01 versus PBS.

To characterize the pathway underlying the effect of Pir-B, IL-6 was administered at the optimal dose (20 ng/ml) in a co-culture of CD4^+^ T cells and DCs isolated from 5-week tumor-bearing mice. The percentage of Th17 cells and the level of IL-17 were increased in IL-6 group compared with PBS group, whereas there was a significant decrease in the percentage of Tregs (Figure 9).

**Figure 10:**
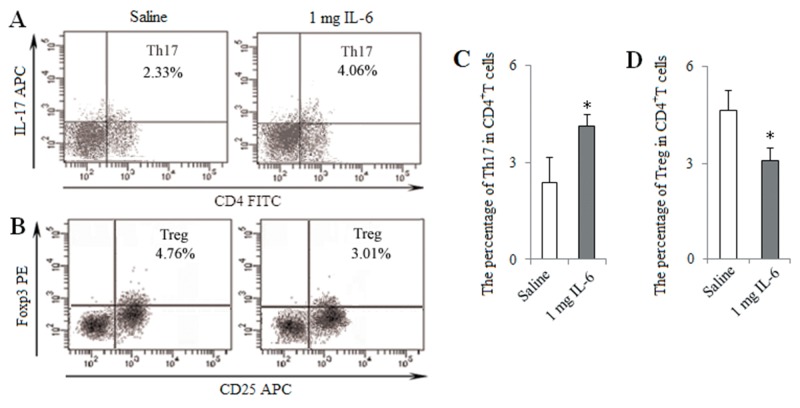
*In vivo* injection of IL-6 induced Th17 response and inhibited Treg differentiation Tumor-bearing mice were treated with intraperitoneally injection of IL-6 (1 mg per mice) or saline (0.1ml) as control at 1, 2 and 3 weeks after LLC inoculation. The percentages of tumor infiltrating Th17 **(A)** and Treg **(B)** cells were analyzed by flow cytometry. The percentages of Th17 **(C)** and Treg **(D)** cells were shown in the histogram. ^*^P<0.05, ^**^P<0.01 versus Saline.

For further determine the role of IL-6 *in vivo*, tumor-bearing mice were treated with intraperitoneally injection of IL-6 (1 mg per mice) at 1, 2 and 3 weeks after LLC inoculation. The percentage of Th17 cells was increased in IL-6 group compared with Saline group, whereas the percentage of Tregs was decreased (Figure 10). These results suggest that Pir-B inhibit Th17 response and promote Treg differentiation via IL-6 pathway.

## DISCUSSION

The tumor related immunosuppression makes the ability to monitor tumor escape and metastasis weak. The immunosuppressive mechanism underlying the progression of lung cancer has not yet been fully elucidated. In the present study, the DC function and Th17/Treg balance are impaired during the progression of lung cancer, and Pir-B is involving in inhibiting the DC function and Th17 response and promoting Treg differentiation via IL-6 pathway. Our study has demonstrated that Pir-B inhibits the DC function and disturbs the Th17/Treg balance via IL-6 pathway, contributing to inhibited antitumor immunity of lung cancer.

Tregs depress the antitumor immune response, depending on the secretion of TGF-β and IL-10 [21], whereas the role of IL-17 in tumor immunity remains fully undefined. In the present study, the expression of Th17 cells and related IL-17 was decreased, whereas the expression of Tregs and related TGF-β was increased, suggesting that the Th17/Treg balance was disturbed during the progression of lung cancer. It was supported by the study [22] that there was a significant decrease in the expression of Th17 cells and IL-17 and an increase in the percentage of Treg in peripheral blood of lung cancer patients. Similar results were obtained by Zhang [23] that the Th17/Treg ratio decreased in lung cancer patients. Above findings confirm a novel mechanism in which lung cancer may occur as a consequence of the Th17/Treg imbalance, suggesting that Th17 cells and Tregs play important roles in the lung cancer pathogenesis [21, 22]. This information provides important insights into understand the immunosuppressive mechanism underlying the development of lung cancer.

DCs induce the differentiation of CD4^+^ T cell subsets, and the immune function of DCs rely mainly on the specific receptors on their surfaces [24–26]. Pir-B plays an inhibitory role in the cross talk of DCs and CD4^+^ T cells [27, 28]. Increased expression of Pir-B inhibits the DC maturation and Th1 cell response, and promotes Th2 cell differentiation [20]. However, little is known about the role of Pir-B involved in the differentiation of Th17 cells and Tregs in lung cancer pathogenesis. In the present study, the expression of Pir-B was increased, and this was accompanied by an inhibited function and maturation of DCs. After transfection with Pir-B siRNA, the inhibited function and maturation of DCs were restored, and meanwhile Th17 response was increased, whereas Treg differentiation was decreased. Our findings therefore suggest that Pir-B disturbed the Th17/Treg balance by inhibiting the function and maturation of DCs during the progression of lung cancer.

Activated CD4^+^ T cell subset differentiation is strictly controlled by cytokine environments [29]. IL-6 is necessary for DC-dependent Th17 cell differentiation and IL-17 production [30]. IL-6, in turn, inhibits the development of Tregs, suggesting that Th17 cells and Tregs arise in a mutually exclusive fashion [31]. Recent report has suggested that different DCs produce different cytokine that may direct CD4^+^ T cell subset differentiation [32]. In the present study, IL-6 secreted by DCs was decreased during the progression of lung cancer, and this was accompanied by the decreased response of Th17 cells and the increased differentiation of Tregs. Furthermore, IL-6 level was up-regulated after transfection with Pir-B siRNA, and Th17 response was increased, and Treg differentiation was decreased. And the administration of IL-6 *in vitro* and *vivo* increased also Th17 response, and decreased Treg differentiation. These results were supported that the inhibited Th17 response and increased Treg differentiation were owing to the decreased IL-6 secreted by DCs [33, 34].

In conclusion, the present study describes a novel immunosuppressive mechanism involved in the progression of lung cancer. Pir-B impaired the DC function and disturbed the Th17/Treg balance via IL-6 pathway during the progression of lung cancer, contributing to tumor related immunosupression. It appears important to elucidate the immunosuppressive mechanism underlying the development of lung cancer.

## MATERIALS AND METHODS

### Cell lines and animals

Lewis lung cancer (LLC) tumor cell line was purchased from Shanghai Institutes for Biological Sciences, Chinese Academy of Sciences and maintained in the Department of Immunology, Harbin Medical University (Harbin, China). Tumor cells were cultured in complete RPMI 1640 medium (Invitrogen, Carlsbad, CA) supplemented with 10% FBS, 100 U/ml penicillin and 100 g/ml streptomycin in a humidified cell incubator with an atmosphere of 5% CO_2_ at 37°C.

Female C57BL6/J mice (Vital River Laboratories, Beijing, China) at 6-8 weeks old were acclimated under specific-pathogen-free conditions. All protocols involving animals were approved by the local Animal Research Ethics Committee of Harbin Medical University (Harbin, China). LLC tumor cells (1×10^6^) were resuspended in 100 μl PBS and subcutaneously inoculated into the right flanks of mice to initiate tumor [35]. Tumor-bearing mice were divided into four groups (LLC week 1, 3, 5 and 7 groups, n=20/group). Tumor-free mice were considered as Control (n=20). All mice were anesthetized by 2% sodium pentobarbital, and tumor tissues were removed and incubated in digestion buffer (RPMI medium containing 5% fetal bovine serum, 0.02% collagenase IV, 0.002% DNase I, and 10 U/ml of heparin). After trypsinization, tissues were filtered through 300 molybdenum sieving mesh to obtain single cell suspensions to assay the percentages of Th17, Tregs and DCs.

### Magnetic separation and culture

Spleen tissues were removed and splenic lymphocytes were isolated by the lymphocytic separation medium. Splenic lymphocytes were resuspended in MACS buffer and incubated with anti-CD4 and anti-CD11c magnetic beads at 4°C for 15 min. After washing, CD4*^+^* T cells and CD11c^+^ DCs were positively selected by applying splenic lymphocytes onto MS columns that were placed on a MACS separator (all from Miltenyi Biotec, Auburn, CA). The purity of CD4*^+^* T cells and DCs was greater than 90%. Purified CD4*^+^* T cells were cultured in the presence of 30 ng/ml anti-CD3, 30 ng/ml anti-CD28, and 20 μg/ml rIL-2 for 48 h. Purified DCs were cultured in the medium (complete RPMI 1640 medium with 20 ng/mL rmGM-CSF and 20 ng/mL rIL-4) for 48 h.

### *In vitro* cytotoxicity assessment

*In vitro* cytotoxicity of splenic lymphocytes were estimated with the CytoTox 96® Non-Radioactive Cytotoxicity Assay (Promega, Madison, WI, USA). Splenic lymphocytes were incubated with LLC cells (2×10^5^/well) at different effector/target ratios (25:1, 50:1, and 100:1) in the 96-well culture plates for 4 h. The supernatants were collected and the absorbance at 490 nm was tested to evaluate the lactate dehydrogenase (LDH) [36]. The percentage of LDH release was calculated by the following formula: % Cytotoxicity = (Experimental – Effector Spontaneous – Target Spontaneous)/(Target Maximum – Target Spontaneous) ×100.

### siRNA transfection

Twenty-four h before siRNA transfection, purified DCs from spleen of 5-week tumor-bearing mice were seeded in 24-well plates at 0.5×10^5^ cells/well in Opti-minimal essential medium (Opti-MEM, Invitrogen, USA). Cells were seeded in a 24-well plate and cultured to 30-50% confluency at the time of transfection. Transfection of siRNA molecule was performed using the Silencer^TM^ siRNA construction kit with a final concentration of 200 nmol/L using Lipofectamine 2000 (Invitrogen) according to the manufacturer’s instructions.

DCs transfected with FITC-Oligo served as positive control to detect the transfection efficiency. The negative control (NC) siRNA was used as blank control. The forward specific sequence for Pir-B was 5′-AUUGAGAGCCUUCAGGUACAUAUGC-3′, and the reverse sequence was 5′-GCAUAU BGUACCUGAAGGCUCUCAUU-3′.

For determine the role of Pir-B *in vitro*, purified CD4^+^ T cells from spleen of 5-week tumor-bearing mice were co-cultured with DCs or DCs transfected with Pir-B siRNA at 10:1 ratio, 2.5 μg/ml anti-CD28 mAb in anti-CD3 mAb-coated (5 μg/ml) 96-well plates for 48 h.

For determine the role of IL-6 *in vitro*, purified CD4^+^ cells from spleen of 5-week tumor-bearing mice were co-cultured with DCs at 10:1 ratio, 2.5 μg/ml anti-CD28 mAb in anti-CD3 mAb-coated (5 μg/ml) 96-well plates in the presence of IL-6 at the optimal dose (20 ng/ml) or PBS (0.1ml) as control for 48 h.

For further determine the role of Pir-B *in vivo*, DCs transfected with Pir-B siRNA (1 × 10^6^ cells per mice) were transferred into recipient animals by tail vein injection at 1, 2 and 3 weeks after LLC inoculation. Saline (0.1ml) was considered as control. Tumor growth was monitored once weekly, and tumor volume was assessed by measuring tumor size with digital calipers using the following formula: volume = 0.52 × (longth × width^2^). Ten mice from each group were randomly selected for the survival rate analysis. Kaplan-Meier curve of survival as defined as the date mice were sacrificed either because there tumors were larger than 1.5 cm^3^ or they had ulcerated tumor requiring sacrifice in accordance with ethical standards.

For further determine the role of IL-6 *in vivo*, mice were intraperitoneally injection of IL-6 (1 mg per mice) or saline (0.1ml) as control at 1, 2 and 3 weeks after LLC inoculation. The percentages of tumor infiltrating Th17 and Tregs were assayed.

### Flow cytometry analysis

After cell density was adjusted to 1×10^6^/mL, 100 μL single cell suspensions were added into flow tube and incubated with FITC-labeled anti-CD4, APC-labeled anti-CD25 and anti-IL-17, PE-labeled anti-Foxp3, FITC-labeled anti-CD11c, MHC-II, CD80, CD86 and Pir-B mAb (all from BD Pharmingen) for 30 min at 37°C. To detect IL-17^+^ or Foxp3^+^ cells, these cells were fixed and permeabilized. The appropriate fluorescein-conjugated IgG were used as isotype control. Cells were analyzed using a BD LSRII flow cytometer (Becton Dickinson, San Diego, CA) on at least 5,000-10,000 events.

### Real-time PCR analysis

Tumor tissues were homogenized using tissue grinders, and total RNA was extracted using TRIzol reagent (Invitrogen). The total RNA (1 μl) was reverse-transcribed to cDNA, and 20 μl of the reverse transcription reaction system was placed in 42°C water for 60 min and heated in 70°C water for 15 min to deactivate the reverse transcriptase. The primers were designed and synthesized at the Shanghai Institute of Biochemistry (China). The following primer pairs were used: Pir-B, Forward: 5′-GTCTGTGGCCTTCATCCTGTTC C-3′ and Reverse: 5′-TGTTCAGCTCCACTCCATCCTCAG-3′ (Invitrogen). The amplification reaction underwent 30 cycles of degeneration at 95°C for 1 min, annealing at 58.5°C for 1 min, and extension at 72°C for 1 min. A final extension was performed at 72°C for 10 min. Polymerase chain reaction amplification was performed on a DNA Engine thermal cycler (Bio-Rad, Hercules, CA) using SYBR Green I (Invitrogen). All samples were assayed in triplicate for each experiment. The gene expression were calculated using the ΔΔCq method with ABI Prism® 7900HT software, and the values were expressed as 2^−ΔΔCq^. The relative fold changes of the target gene expression were normalized against GAPDH.

### Western blotting analysis

Single cell suspensions from tumor tissues were lysed in 100 μl of lysis buffer on ice for 30 min and the supernatants were collected by centrifugation at 4°C and 14,000 r/min. Total protein was extracted and the protein concentration was determined with the Bradford method. A 100-μg quantity of protein was subjected to 10% SDS-polyacrylamide gel electrophoresis and electrotransferred to a polyvinylidene difluoride membrane. The membrane was blocked for 1 h at room temperature and incubated with antibody against Pir-B or β-actin (1:1,000 dilution, Santa Cruz, CA) at 4°C overnight. The membranes were incubated with an IRDye800-conjugated secondary antibody (Biotrend Chemikalien GmbH, Köln, Germany). The protein-antibody complexes conjugated to IRDye800 were visualized with an Odyssey Infrared Imaging System (LI-COR Biosciences, Bad Homburg, Germany). The protein relative quantity was determined as the ratio to β-actin.

### ELISA

The levels of IL-17, TNF-β, IL-6, IL-10 and IL-12 in the homogenate of tumor tissues and the supernatants of the co-culture system were measured using Cytometric Bead Array (CBA) inflammation kit (BD Biosciences, San Diego, CA).

### Statistical analysis

Power analysis was based on the results of preliminary experiments comparing immune variables among groups and yielded a sample size of n=20 (α=0.05; 1–β=0.9) per group. The data were analyzed with SPSS 13.0 software (serial 5031432, Stats Data Mining Co., China) and are presented as the means ± SD. Western blot densities were analyzed with the Kruskal-Wallis test followed by Dunn’s post-hoc test. Survival of mice was estimated using the Kaplan-Meier method. Other data were subjected to one-way analysis of variance (ANOVA) followed by a Bonferroni correction for a post-hoc t test. *P*<0.05 was considered significant.

## Abbreviations

Tregs: regulatory T cells
DCs: dendritic cells
imDCs: immature DCs
mDCs: mature DCs
APCs: antigen presenting cells
Pir-B: paired immunoglobulin like receptor B
